# Rheological Droplet Interface Bilayers (rheo-DIBs): Probing the Unstirred Water Layer Effect on Membrane Permeability via Spinning Disk Induced Shear Stress

**DOI:** 10.1038/s41598-017-17883-0

**Published:** 2017-12-14

**Authors:** Nathan E. Barlow, Guido Bolognesi, Stuart Haylock, Anthony J. Flemming, Nicholas J. Brooks, Laura M. C. Barter, Oscar Ces

**Affiliations:** 1 Department of Chemistry, Imperial College London, Exhibition Road South Kensington, London, SW7 2AZ UK; 2 Institute of Chemical Biology, Imperial College London, Exhibition Road South Kensington, London, SW7 2AZ UK; 3 Department of Chemical Engineering, Loughborough University, Loughborough, Leicestershire LE11 3TU UK; 4 Syngenta, Jealott’s Hill International Research Centre, Bracknell, Berkshire RG42 6EY UK

## Abstract

A new rheological droplet interface bilayer (rheo-DIB) device is presented as a tool to apply shear stress on biological lipid membranes. Despite their exciting potential for affecting high-throughput membrane translocation studies, permeability assays conducted using DIBs have neglected the effect of the unstirred water layer (UWL). However as demonstrated in this study, neglecting this phenomenon can cause significant underestimates in membrane permeability measurements which in turn limits their ability to predict key processes such as drug translocation rates across lipid membranes. With the use of the rheo-DIB chip, the effective bilayer permeability can be modulated by applying shear stress to the droplet interfaces, inducing flow parallel to the DIB membranes. By analysing the relation between the effective membrane permeability and the applied stress, both the intrinsic membrane permeability and UWL thickness can be determined for the first time using this model membrane approach, thereby unlocking the potential of DIBs for undertaking diffusion assays. The results are also validated with numerical simulations.

## Introduction

**Figure 1:**
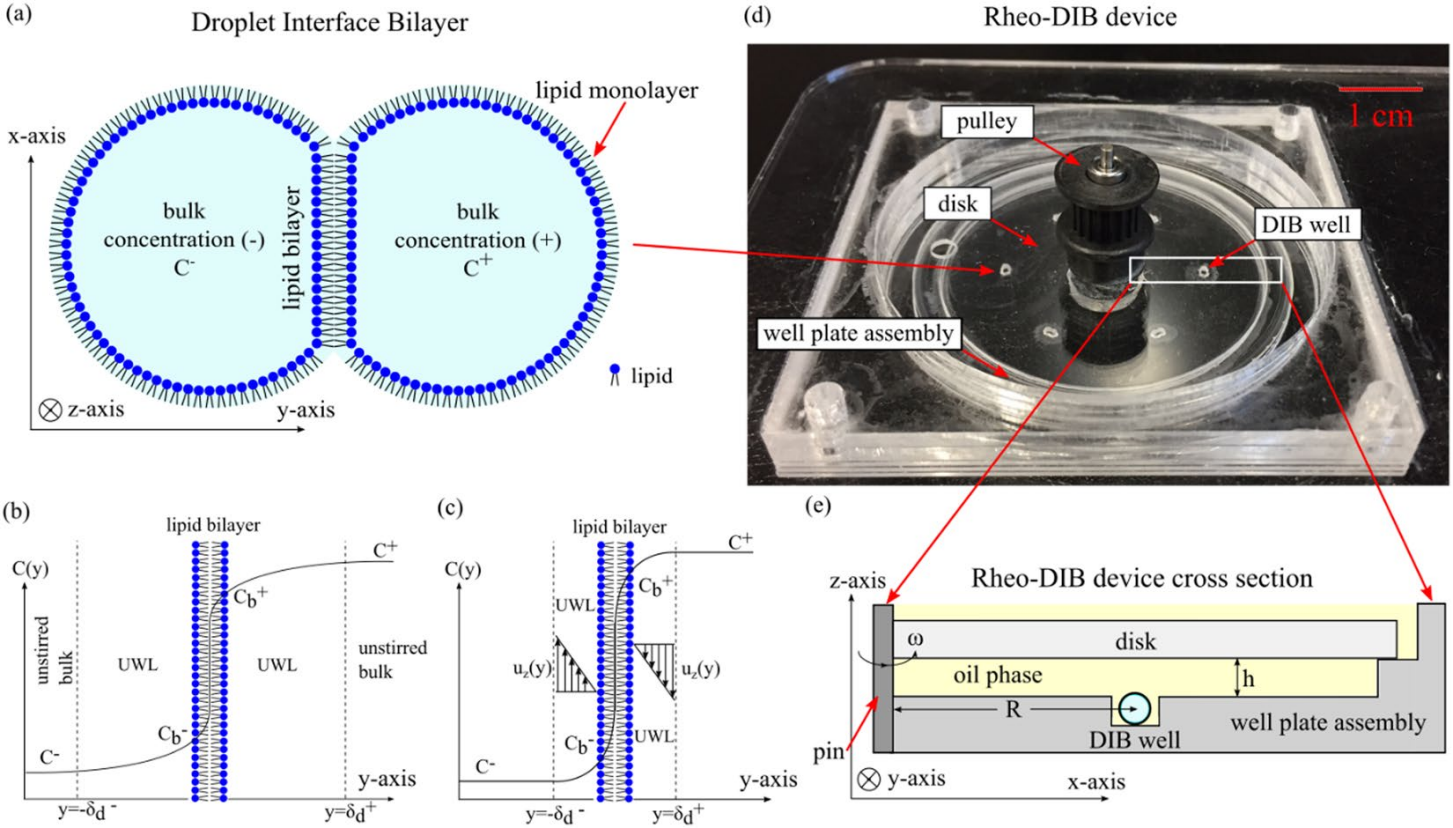
Cartoon of droplet interface bilayer (DIB) (**a**) that consists of two aqueous lipid-emulsions droplets that have formed a lipid bilayer membrane at the interface. Sketch (**b**) of a species concentration profile across a lipid bilayer membrane (y-axis origin is on the membrane) affected by a diffusional UWL of thicknesses ${{\rm{\delta }}}_{{\rm{d}}}^{\,\pm }$ where a solute in the bulk solution C^+^ on the (+) side permeates across the membrane and water layers to the (−) side bulk solution of concentration C^−^. Note that the bilayer membrane concentration differs on either side by ${{\rm{C}}}_{{\rm{b}}}^{+}$ and ${{\rm{C}}}_{{\rm{b}}}^{-}$. Sketch (**c**) of a species concentration profile across a lipid bilayer membrane with a concentration gradient on opposing sides C^+^ and C^−^, where a parallel fluid flow in the bulk solution develops a laminar boundary layer at the interface. This fluid dynamic boundary layer affects the bilayer concentrations (${{\rm{C}}}_{{\rm{b}}}^{+}$ and ${{\rm{C}}}_{{\rm{b}}}^{-}$) and the diffusional UWL thickness ${\,{\rm{\delta }}}_{{\rm{d}}}^{\,\pm }$ as a function of fluid velocity u_z_(y) in the z direction. Note that the thicknesses are not drawn to scale. Photograph (**d**) of rheo-DIB chip which can be assembled on a fluorescence microscope. A non-polar shearing media (hexadecane) is filled to 3 mm above the disk in the well plate assembly and the DIBs are formed within the DIB wells. The disk is driven by DC brushed motor and a toothed band/pulley up to 200 RPM, the maximum speed in which DIBs are stable for this configuration. Cartoon (**e**) of an axial-radial (x, z) cross section of rheo-DIB chip where the aqueous DIB sits in a confinement DIB well (at R = 17 mm) which is surrounded by an oil phase, and is exposed to shear stress by a disk spun about the z-axisat a determined height (h = 1 mm)above the top of the droplets and angular velocity ω.

Droplet interface bilayers (DIB)^1,2^ are model lipid membranes which are formed upon contact of two lipid monolayers at the interface of water droplets immersed in a second immiscible phase (Fig. 1a). Recently, DIBs have emerged as a powerful tool for assembling model membranes and, among other uses, they have been applied to assaying membrane permeability^3,4^. DIBs appear well suited to this application as they typically consist of two small volume (<10 µL) aqueous compartments that allow for loading test solutes at a specified concentration gradient, where the bulk concentrations in the droplets are C^+^and C^−^ respectively. By monitoring the bulk droplet concentrations over time the bilayer permeability can be determined as the ratio of the permeate flux J across the membrane of thickness δ_m_ and the droplet concentration difference ΔC = C^+^−C^−^. However, as shown in Fig. 1b, there exists regions of thickness ${{\rm{\delta }}}_{{\rm{d}}}^{+}$ and ${{\rm{\delta }}}_{{\rm{d}}}^{-}\,\,$adjacent to either side of the interface of the permeating membrane where the solute concentration departs from the bulk value, thereby causing an additional resistance to mass transfer from the membrane to the bulk. Due to the presence of these regions, also known as ‘unstirred water layer’ (UWL)^5^ or ‘diffusional boundary layer’^6,7^ the lipid bilayer is exposed to a reduced concentration difference ${{\rm{\Delta }}C}_{{\rm{b}}}={{\rm{C}}}_{{\rm{b}}}^{+}-{{\rm{C}}}_{{\rm{b}}}^{-}$, where ${{\rm{C}}}_{{\rm{b}}}^{+}$ and ${{\rm{C}}}_{{\rm{b}}}^{-}$ are the solute concentrations on either side of the bilayer, hence reducing the effective flux J. As a consequence, the measured quantity J/ΔC provides only an effective value of permeability, which is systematically lower than the intrinsic membrane permeability given by J/ΔC_b_. The effect can be attenuated by mixing of the bulk solution (advection) and shearing of the fluid adjacent to the membrane as shown in Fig. 1c. This shearing is modelled by a fluid mechanics analogue to the UWL called the Blasius laminar flow boundary layer^8^ which is a function of the velocity parallel to the membrane in the z direction u_z_(y), a phenomenon first introduced by Prandtl^9^. From this shearing we see the regions of thickness ${\delta }_{d}^{\pm }\,\,$adjacent to the interface decrease in size.

The effect of the UWL on membrane permeability is a well-documented phenomenon^5^. It was first noted in the biological community by Dainty in 1963 and 1966 with the analysis of the osmotic permeability of oleic acid monolayers, plant cells and frog skin pores^10–13^. Since then, the UWL has been studied in the permeability of erythrocytes, intestine, and gallbladder epithelial cells^14–26^. In addition to passive transport, evidence is given that supports the supposition that the UWL affects active transport kinetics^27^. The range of sizes of biological and artificial membrane UWL thicknesses are as low as 5 µm for water flux across blood cells, 40 µm for water flux across gallbladder epithelium, and UWL thickness on the order of a ~100 µm for ions permeating artificial planer bilayers^14,26,28^. In DIB systems, the UWL effect is exacerbated by the fact that permeability measurements are performed in stagnant conditions under which the UWL thickness is at its maximum value. It is worth noting that a small membrane permeable molecule with a typical diffusion coefficient of D = 5 × 10^−6^ cm^2^ s^−1^ would take approximately 10 minutes to diffuse across a 0.1 µL droplet, assuming the timescale ${\rm{\tau }}\approx \frac{{{\rm{d}}}^{2}}{{\rm{D}}}$ is given by the droplet diameter d and diffusivity D. This characteristic time τ can be similar to the permeability assay time^3,4^ hence suggesting that the mass transfer resistance of the membrane is comparable to the resistance of the UWL. Therefore, neglecting the UWL effect in DIB systems, assembled with sub-µL or larger volume droplets, will result in significant systematic errors on membrane permeability measurements. Unfortunately, accounting for the UWL effect is not straightforward as such previously published DIB based systems have not explored the effect, which is a necessity if the intrinsic rather than effective membrane permeability is to be obtained.

To investigate and quantify the UWL effect in DIB systems we developed the rheological droplet interface bilayer (rheo-DIB) chip (Fig. 1d). This device has the capability to expose DIBs to well controlled shear stresses (see ESI), hence enabling a wide range of applications in bio-membrane science. More specifically, we have developed a method for combining controlled DIB formation and stirred permeability assays with a spinning disk that induces shear on the droplets and membrane surfaces. The DIB is formed inside a laser cut well filled with a non-polar shearing phase (such as hexadecane) that will contain the droplets and prevent movement even under shear (Fig. 1e). The fluid motion imparts shear on the DIBs and ultimately internal circulation inside the droplets. Rotating disks have been used previously to modify the unstirred diffusion layer to improve dissolution^29^ as well as to apply shear stress to cultured endothelial cells^30^. It has also been suggested by Pedley to employ rotating disks to measure UWL thicknesses^5^. Due to the fact that rotating disk fluid flows are well studied (and are relatively simple to manufacture) it is chosen over the alternative techniques such as magnetic stirring bars or flow channels. A critical advantage of a spinning disk type chip for shear stress experimentation is that the disk may be removed and replaced rapidly for assaying a range of samples. Furthermore, compared to microfluidic approaches the rheo-DIB chip allows for a higher level of flexibility which can control shear by radial position, disk height, and rotation speed, whereas microfluidic channels have a fixed geometry.

In this study, the rheo-DIB chip was adopted to probe for the first time the UWL effect in DIB systems under both stagnant and stirring conditions, and hence, to determine the effective as well as intrinsic membrane permeability. Our investigation demonstrates that for the small molecule (resorufin) permeating lipid membrane connecting sub-µL droplets, the intrinsic membrane permeability can be as large as twice the measured effective permeability. Consequently, the latter quantity, which depends on system parameters including droplet size and liquid viscosity, can no longer be considered a reliable parameter for the characterisation of the membrane properties unless the effect of the UWL is accounted for. Note that resorufin was chosen as the model molecule as it has a strong and linear fluorescent signal with respect to concentration, it is highly permeable relative to other fluorescent dyes, is a low-cost and commercially available molecule, and finds widespread use in biological assays such as cell viability.

## Results

### The rheo-DIB device

The current modern artificial cell permeability technique, the parallel artificial membrane permeability assay (PAMPA)^31^, does not take into account the UWL directly, but requires the use of iso-pH mapping and pK_a_ flux assays to estimate the UWL effect^32^. Though the 96-well plate PAMPA technique is high-throughput, stirring the system directly can often be a drawback and requires specialized kit^33^, or a large quantity of small stirring bars; undoubtedly, stirring and mixing are not trivial problems in microfluidic platforms^34^. Our spinning disk chip introduces the ability to mix the permeating system lost to the current technology. The device is of relatively simple design and is fashioned with layers of laser cut poly(methyl methacrylate) (PMMA). A disk is applied at height h = 1 mm above the DIB well and is spun to induce flow in the fluid region below the disk. The DIB is located at a radial distance of R = 17 mm from the disk axis. The disk is spun with a brushed DC motor that is controlled by a variable power supply. The 0.8 μL droplet pairs sit in cylindrical cavities engraved in the well plate assembly, and are immersed in the oil-filled region between the rotating disk and the base. More information is provided in the Methods section.

### Flow profile in the sheared DIBs

**Figure 2:**
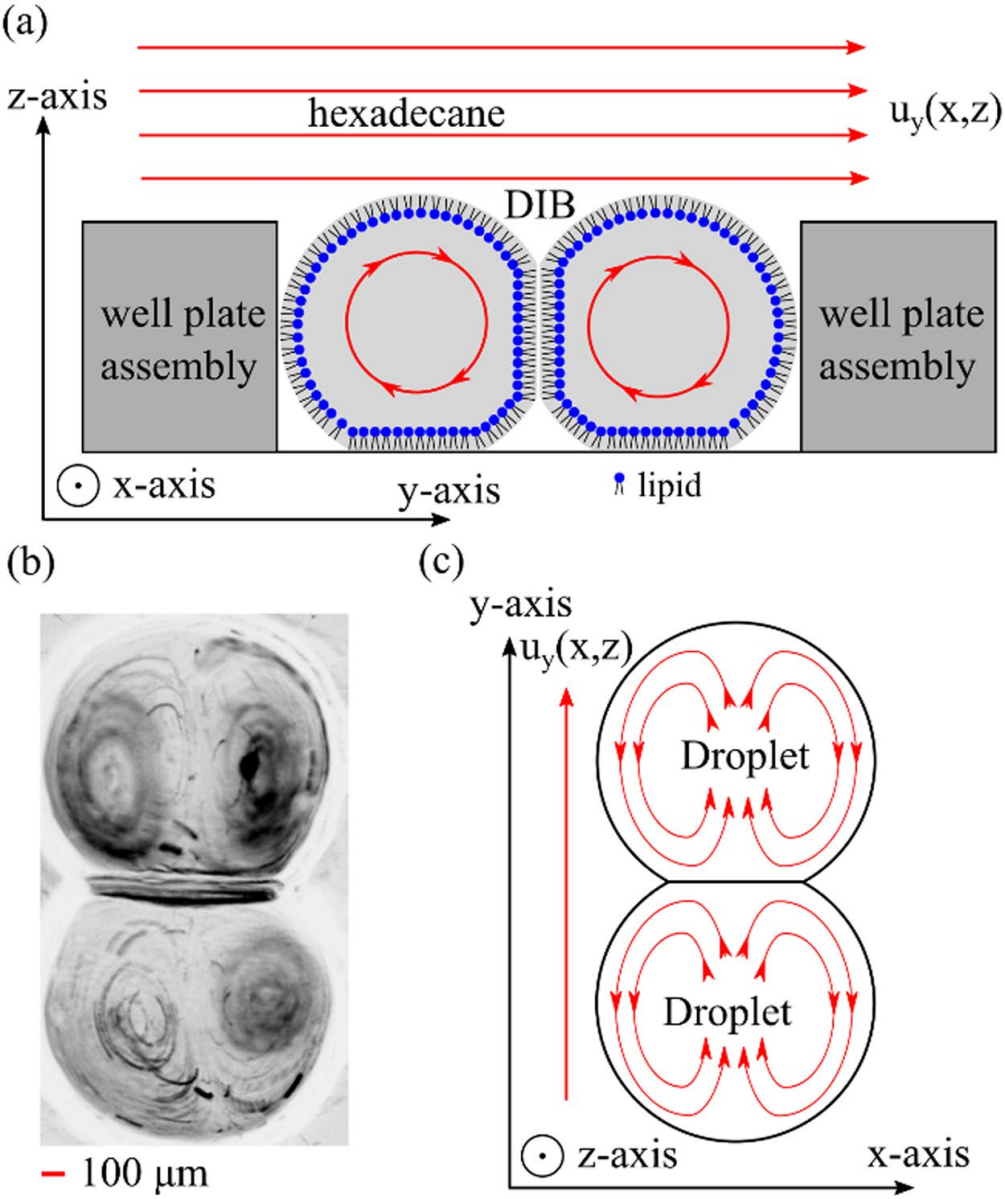
Cartoon (**a**) of axial-azimuthal (y, z) cross-section with streamlines of recirculation flow in a DIB pair due to shearing flow at the top of the droplets from the non-polar phase (hexadecane) with the velocity flow profile u_y_ (x, z). Micrograph (**b**) of a ‘minimum intensity Z-projection’ (processed on ImageJ) from a (top view) image sequence of particle motion in a double lobed, symmetric vortex pair within a DIB. Cartoon (**c**) of the streamlines of the top half of the DIB pair on the x-y plane in (**b**), which shows the flow direction and recirculation flow around the side of the droplets.

Controlled shear stresses are applied to the lipid membranes by the disk above the droplets and rotating at constant angular velocity ω. The working ranges are 0 to 200 rotations per minute (RPM). The spinning disk induces circular flow in the DIBs, as exemplified by the streamlines in Fig. 2a. Note that by having a set of identical DIB pairs located at different radial distance (in this case 17 mm) a range of shear stresses can be tested simultaneously at a constant disk speed. This is an advantage over microfluidic devices that involves modifying the flow or the viscosity to adjust the shear stress which requires time for flow stabilization. The induced flow and vorticity inside the DIB is clearly shown in Fig. 2b from a Z-projection of 50 images captured over a 1 second period under shear stress (~100 RPM). The flow profile can be captured by loading the droplets with 5 µm polystyrene latex beads at approximately 10^6^ particles mL^−1^ ^35^. Due to the spherical shape of the droplets, the vorticity patterns show the (non-toroidal) circulation of fluid around the side of the droplets in Fig. 2c. This dipole vortex is verified using COMSOL Multiphysics models and has been reported previously for vesicle systems in both simulations and experiments^36,37^.

### Effective, intrinsic, and unstirred water layer permeability

The effective permeability P_eff_ of a membrane to a solute is defined as the overall permeability from one side of the UWL at ${\rm{y}}=-{{\rm{\delta }}}_{{\rm{d}}}^{-}\,,$through the membrane, and across the opposing UWL at ${\rm{y}}={{\rm{\delta }}}_{{\rm{d}}}^{+}$ as shown in Fig. 1b for stagnant conditions and Fig. 1c for stirred conditions. This implies that the effective permeability is a function of the UWL permeability P_UWL_ and of the intrinsic membrane permeability P_m_. The value of P_eff_is measured directly as a function of diffusion rate k, droplet volume V, and interface area A by equation (1) ^4^.

1 $${{\rm{P}}}_{{\rm{eff}}}=\frac{{\rm{kV}}}{{\rm{A}}}$$

we can also define the UWL permeability^5^ for a single layer as,

2 $${{\rm{P}}}_{{\rm{UWL}}}=\frac{{\rm{D}}}{{{\rm{\delta }}}_{{\rm{d}}}^{\pm }\,}$$

which is a function of the bulk diffusivity D and the UWL thicknesses ${{\rm{\delta }}}_{{\rm{d}}}^{\pm }$ . The parameter ${{\rm{\delta }}}_{{\rm{d}}}^{\pm }$ can be rigorously defined in terms of the bulk concentrations C^±^, the concentrations at either side of the membrane ${{\rm{C}}}_{{\rm{b}}}^{\pm }$, and the concentration gradients at the membrane $\,{\frac{\partial {\rm{C}}}{\partial {\rm{y}}}|}_{{\rm{y}}={0}^{\pm }}\,\,$ by^5^,

3 $${{\rm{\delta }}}_{{\rm{d}}}^{\pm }=\,\frac{{{\rm{C}}}^{+}-{{\rm{C}}}_{{\rm{b}}}^{+}}{{\frac{\partial {\rm{C}}}{\partial {\rm{y}}}|}_{{\rm{y}}={0}^{+}}}=\frac{{{\rm{C}}}_{{\rm{b}}}^{-}-{{\rm{C}}}^{-}}{{\frac{\partial {\rm{C}}}{\partial {\rm{y}}}|}_{{\rm{y}}={0}^{-}}}$$

The (lumped) intrinsic permeability can be similarly defined as^38,39^,

4 $${{\rm{P}}}_{{\rm{m}}}=\frac{{{\rm{D}}}_{{\rm{m}}}}{{{\rm{\delta }}}_{{\rm{m}}}}$$

which is a function of the membrane diffusivity D_m_, and the membrane thickness δ_m_. Note that the true intrinsic membrane permeability ${{\rm{P}}}_{{\rm{m}}}^{\ast }$ is a function of the partition coefficient K, however for simplification we will lump together the terms so that ${{\rm{P}}}_{{\rm{m}}}=\frac{{{\rm{P}}}_{{\rm{m}}}^{\ast }}{K}$. Following the electrical analogy^5^, the serial resistivity to mass transfer is given by equation (5), as a function of the intrinsic membrane permeability P_m_ and the UWL permeability P_UWL_,

5 $$\frac{1}{{{\rm{P}}}_{{\rm{eff}}}}=\frac{1}{{{\rm{P}}}_{{\rm{m}}}}+2\frac{1}{{{\rm{P}}}_{{\rm{UWL}}}}$$

If it is assumed that the UWL has the same thickness on both side of the membrane, namely ${{\rm{\delta }}}_{{\rm{d}}}^{+}={{\rm{\delta }}}_{{\rm{d}}}^{-}={{\rm{\delta }}}_{{\rm{d}}}$, then equation (2) can be simplified as ${{\rm{P}}}_{{\rm{UWL}}}=\frac{{\rm{D}}}{{{\rm{\delta }}}_{{\rm{d}}}}$. Expression (5) can be re-arranged so that the UWL thickness δ_d_ can be expressed as in equation (6) in terms of the intrinsic and effective membrane permeabilities^5^.

6 $${{\rm{\delta }}}_{{\rm{d}}}=\frac{{\rm{D}}}{2}(\frac{1}{{{\rm{P}}}_{{\rm{eff}}}}-\frac{1}{{{\rm{P}}}_{{\rm{m}}}})$$

In this study, the effective permeability measurements were performed by using experimental techniques established previously^4^. In brief, a DIB is prepared with a source droplet (+) containing a high concentration of the permeating molecule C^+^ and a sink droplet (−) containing a low concentration C^−^ of the permeating molecule. In this experiment resorufin is used as a model molecule, and the fluorescence intensity is tracked dynamically using fluorescence microscopy. See the materials and methods section for more details. Since the concentration is in a range that is linear with respect to intensity, the dynamic intensity can be fit to Fick’s first law of diffusion (a 1st order ODE) by equation (7)^13^, for droplet concentrations C^+^ and C^−^.

7 $$\frac{{{\rm{dC}}}^{+}}{{\rm{dt}}}={\rm{k}}({{\rm{C}}}^{-}-{{\rm{C}}}^{+})$$

Note that the diffusion rate is defined as ${\rm{k}}=\frac{{\rm{A}}}{{\rm{V}}}{{\rm{P}}}_{{\rm{eff}}}$, and to find the effective permeability the diffusion rate must be found along with DIB interfacial area and volume. The droplet volume V is known *a priori* as the droplets are deposited with a precision pipette, and the interfacial area A can be measured optically under the assumption that the membrane retains circularity and axial symmetry. It is possible also that the shearing could cause spontaneous pore formation on the membrane that could affect the permeability measurement, as the pores would decrease the hydraulic resistivity. However, this is unlikely as the droplet interfacial tension is high and thus the membrane surface tension is high. The implication is that any pore formation would be unstable as line tension would not be strong enough to prevent the pore from opening and rupturing the membrane. A control experiment was performed with DIBs where the source droplet was loaded with non-permeable dye calcein, and was sheared at 200 RPM. Note that no leakage across the membrane was observed (see ESI).

### Numerical model

In order to verify the effect of stirring on DIB permeability, a 2D COMSOL Multi-physics model of the flow profile **u** = (u_y_, u_z_) is solved in the (y, z) plane x = R with the incompressible Stokes flow equation,

8 $${\rm{\mu }}{\nabla }^{2}{\bf{u}}+\nabla {\rm{p}}=0$$

along with the divergence,

9 $$\nabla \cdot {\bf{u}}=0$$

The fluid velocity **u** depends on the boundary conditions, system pressure p, and the viscosity μ. The dynamic concentration in the droplets c^±^(y, z, t)are a function of the bulk diffusivity D, and the convection from the Stokes flow. These equations are coupled with the dynamic species advection and diffusion equation,

10 $$\frac{\partial {{\rm{c}}}^{\pm }({\rm{y}},{\rm{z}},{\rm{t}})}{\partial {\rm{t}}}=\nabla \cdot ({\rm{D}}\nabla {{\rm{c}}}^{\pm }({\rm{y}},{\rm{z}},{\rm{t}}))+{\bf{u}}\cdot (\nabla {{\rm{c}}}^{\pm }({\rm{y}},{\rm{z}},{\rm{t}}))$$

where the flux across the membrane is found by equation (11), recall that ${P}_{m}=\frac{{{\rm{D}}}_{{\rm{m}}}}{{{\rm{\delta }}}_{{\rm{m}}}}$.

11 $${\bf{n}}\cdot {\rm{D}}{\rm{\nabla }}{{\rm{c}}}^{\pm }=\pm {P}_{m}({{\rm{C}}}_{{\rm{b}}}^{-}-{{\rm{C}}}_{{\rm{b}}}^{+})$$

Note that at all other surfaces **n** · D∇C^+^ = 0, where **n** is the normal vector and D_m_ is membrane diffusivity. More details of the model are provided in the ESI. It is worth noting that the value of intrinsic permeability *P*_*m*_ is unknown a priori and but it can be inferred from the analysis of the experimental data as detailed in the next section.

**Figure 3:**
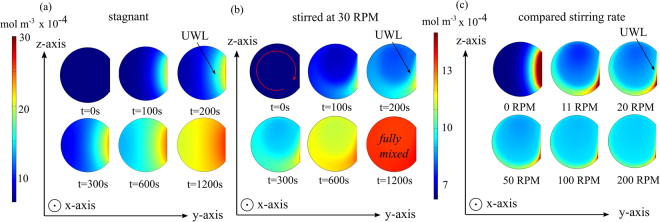
Image (**a**) of a time-dependent droplet concentration c^−^ (y, z, t)profile in a purely diffusional 2D COMSOL model in the y−z plane. In this case the boundary layer is significantly larger and is slower to reach equilibrium. Image (**b**) of a droplet concentration c^−^(y, z, t) profile in a coupled 2D advection-diffusion physics model that is mixed by a spinning disk at 30 RPM. Qualitatively, it is apparent that the Blasius boundary layer decreases the diffusional UWL thickness. Image (**c**) of a droplet concentration c^−^ profile in a coupled 2D advection-diffusion physics model that is mixed by a spinning disk at 0, 11, 20, 50, 100 and 200 RPM (snapshot taken at one minute). Qualitatively, the diffusion layer can be seen to be drastically reduced at higher disk rotation speeds. Note that the droplet diameter is set at 1 mm and that the right side donor droplet (+) is truncated for clarity. An intrinsic membrane permeability of *P*_*m*_ = 1.98 × 10^−4^ cm s^−1^ was used in this model.

The results of the numerical model are shown in Fig. 3 as 2D time-dependent concentration profiles for the acceptor droplet (−). As shown in Fig. 3a under stagnant conditions, a large UWL can be clearly seen extending from the membrane towards the droplet centre. As expected, under stirring conditions (30 RPM), the UWL thickness is significantly reduced (Fig. 3b). The effect of the stirring rate is illustrated further by Fig. 3c, where at one minute simulation time, the UWL thickness can be shown to be sharply affected by the droplet circulation at 11 RPM. The stirring effect on the UWL is less pronounced from 50 to 200 RPM.

### Permeability vs. rotation speed

**Figure 4:**
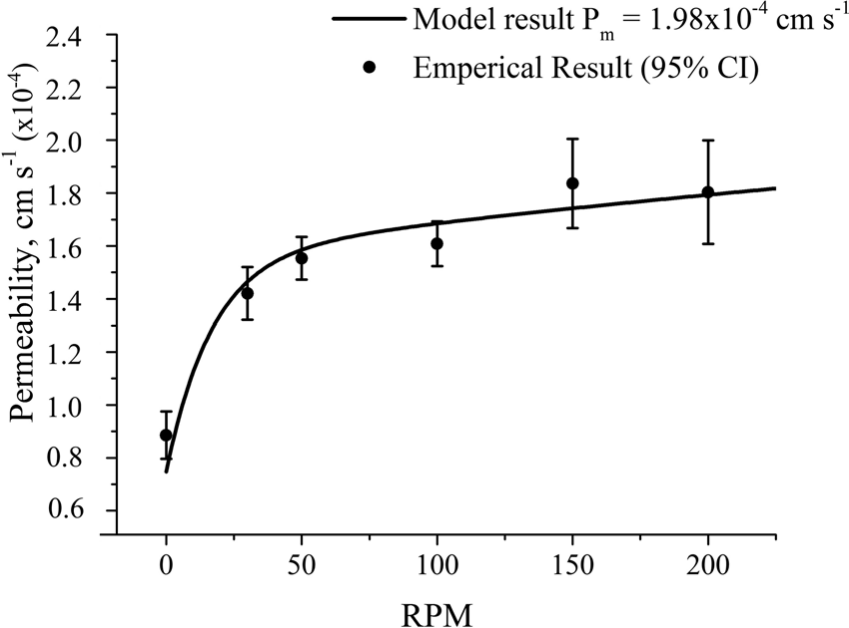
Plot (closed black circles) of measured effective membrane permeability of resorufin in 0.8 μL lipid-in DIBs (DOPC) as a function of disk rotation speed (RPM). The results demonstrate an increase in effective permeability as the mixing and shearing on the membrane decreases the UWL thickness. Sample size n > 10 for each data point error bars set to a confidence interval of 95%. The solid line shows the result from the numerical model best-fitting the experimental data – fitting parameter P_m_ = 1.98 × 10^−4^ cm s^−1^ as demonstrated in the section Permeability VS rotation speed.

A membrane permeability assay was performed on the proof of concept rheo-DIB chip with 0.8 µL droplets loaded with C^−^ = 0.625 and C^+^ = 5.0 μM resorufin at time t = 0. The DIBs were formed of 5 mg mL^−1^ 1,2-dioleoyl-sn-glycero-3-phosphocholine (DOPC) lipid vesicles. Micrograph images of the permeability assay are provided in the ESI. Note that as the droplet concentrations will be uniform at the initiation of the permeability assay, the UWL is expected to take some time before it becomes fully developed, especially for the stagnant case. Therefore the data is analysed only after full development, which implies a good fit to the first order kinetics of Fick’s law of diffusion. The experimental results for effective permeability with respect to disk speed, shown as solid circles in Fig. 4, indicate that by mixing in the laminar flow region the effective membrane permeability P_eff_ first increases significantly with the rotation speed – starting from an unstirred value of (8.86 ± 1.4) × 10^−5^ cm s^−1^– and then it plateaus at higher speeds towards a value of (1.8 ± 0.2) × 10^−4^ cm s^−1^ at 200 RPM. No apparent change in effective permeability at higher speeds suggest that in this regime the UWL thickness is significantly reduced. This is also confirmed by the numerical solutions for the concentration field in the acceptor droplets at increasing rotation speeds (Fig. 3c). As a first approximation, at higher rotation speeds the corresponding UWL effect and the quantitative difference between intrinsic and effective membrane permeability can be neglected. Therefore, the intrinsic membrane permeability P_m_ is estimated to equal the effective permeability at 200 RPM, namely 1.8 × 10^−4^ cm s^−1^. According to equation (6), by using the literature value^40^ for the aqueous diffusivity D of resorufin (i.e. 4.8 × 10^−6^ cm^2^ s^−1^) the UWL thickness is estimated to be δ_d_ ≈ 137(±44) μm in quiescent conditions (*i*.*e*.P_eff_ = 8.86 × 10^−5^ cm s^−1^).

To validate the approximation of a negligible UWL thickness in the high speed regime, an improved estimate of the intrinsic membrane permeability P_m_ can be gleaned from the numerical model. In more details, the P_m_ value enters the simulations as a boundary condition parameter and it can be varied until the model calculates P_eff_ at varying disk rotation speeds that best fit the experimental values. From this optimisation procedure, the intrinsic permeability P_m_ was found to be (1.98 ± 0.19) × 10^−4^ cm s^−1^, which is 10% higher than the original estimate. The corresponding ‘effective permeability vs speed’ curve, obtained from the numerical model and best-fitting the experimental data, is shown as a solid line in Fig. 4. From the numerical calculations, a stagnant UWL thickness of 150 µm and a stirred UWL thickness at 200 RPM of 20 µm are found. These results confirm our assumption that at the highest rotation speed, though it is not null, the UWL thickness is small and the corresponding effective permeability still provides a very good estimate of the actual intrinsic membrane permeability. It also demonstrates that such an estimate can be used to determine the UWL thickness from equation (6) at any rotation speed.

Finally, it is worth noting that stress has been shown to effect membranes properties such as increasing fluidity^41^ and hence permeability^42^. However, in this study the good agreement between experimental and numerical results demonstrates that shear-dependent membrane permeability effect - if at all present – can be neglected to a first approximation.

### The large rheo-DIB device

**Figure 5:**
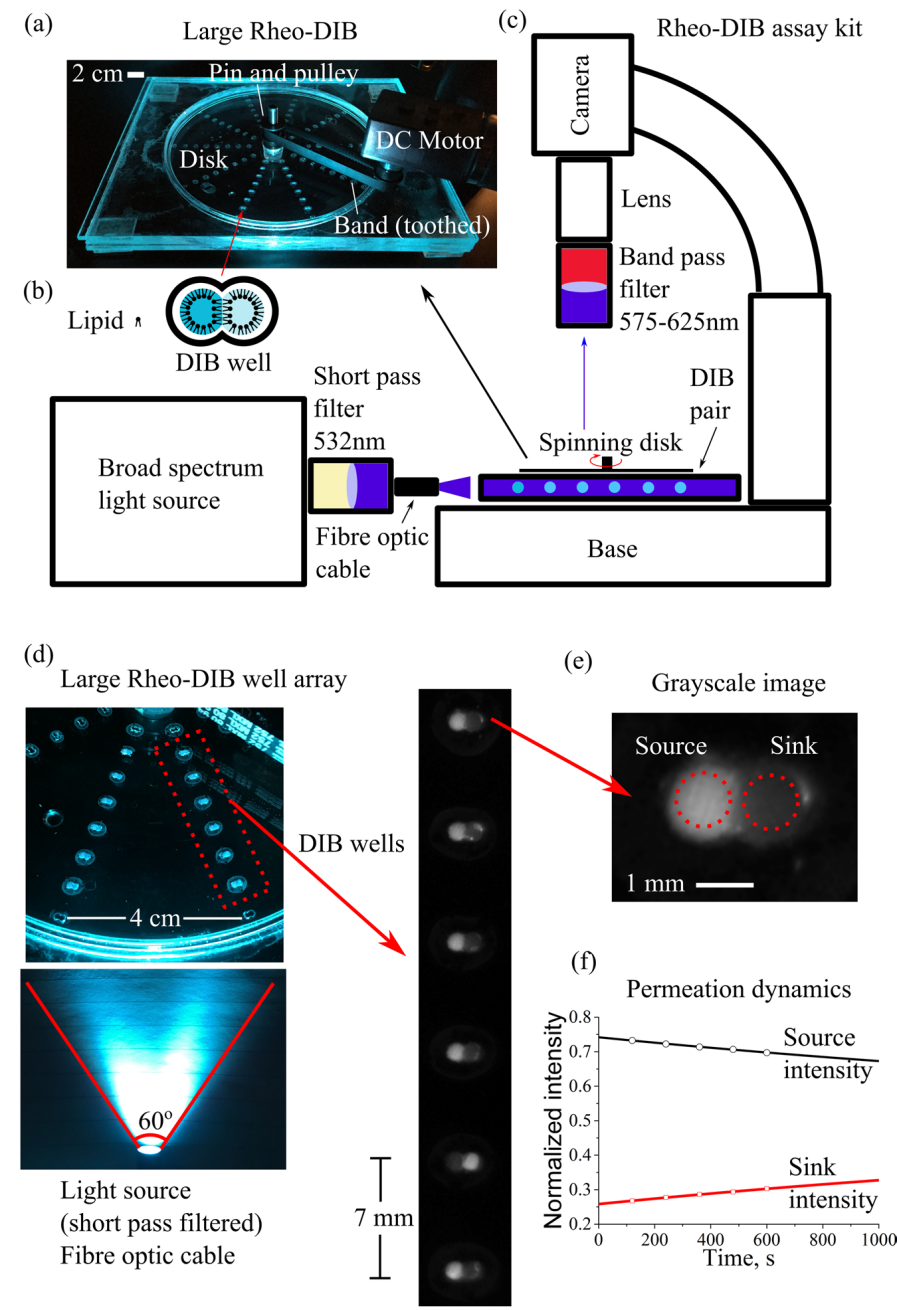
Photograph (**a**) of the large rheo-DIB device for an expanded permeation assay, where 10 columns of 8 DIB wells (**b**) are arrayed around the device centre. The device is driven by a DC brushed motor and variable power supply (20 volts) that is connected to a pulley gear via a toothed band. Schematic (**c**) of the bespoke assay kit consists of an Olympus stereoscope stand with a non-reflective base where the device is mounted below a microscope camera fitted with a macro lens and band pass filters (575–624 nm). The device is illuminated with a broad spectrum light source where the output is filtered by a short pass filter (532 nm). To ensure a uniform illumination, the fibre optic cable position can be adjusted to optimize intensity distribution. Photograph (**d**) of the large rheo-DIB device, where the filtered light illuminates the fluorescent DIBs, which are viewed as micrograph images of the DIB columns. The dynamic intensity of the grayscale DIB images (**e**) are used to measure the permeation dynamics (**f**), which, along with the droplet volume and DIB contact area, are fit to Fick’s 1^st^ law of diffusion.

The proof of concept device was expanded to accommodate an increased number of DIB wells for a high-throughput permeability assay (see Fig. 5a–c). The expanded device is designed to ensure that the permeability assay occurs in a regime where the UWL is minimized and the permeability increase is negligible with respect to increased shear, i.e. at 50 RPM and higher with DIB wells located at a radial distance of 17 mm and higher (as demonstrated in Fig. 4). Therefore the expanded device consists of a 140 mm diameter well plate assembly with 8 DIB wells in a column spaced 7 mm apart, the first of which is located 17 mm from the device centre. Note also that the DIB wells are designed with the same shape configuration as the proof of concept device. Additionally, a 6 mm thick overflow plate is attached to the well plate assembly to prevent oil loss. The 13 mm diameter disk, which sits 1 mm above the DIB wells, is turned on a 3 mm diameter pin and driven by the DC motor.

In this instance, the fluorescent DIBs are illuminated at a 90° angle (see Fig. 5c) with a fibre optic cable. The location of the cone of light can be adjusted by hand until the DIB intensities are uniform. The optimum location (see Fig. 5d) was found to be *ca*. 3.5 cm away from the outer DIB well with a 60° cone of light; with this orientation, the fluorescence intensity of the DIBs across the column deviated by less than 5% (defined by the standard deviation divided by the column average intensity). Note also that negligible photobleaching or dye leakage occurred with this setup for over 2 hours of droplet incubation, with a change in fluorescent intensity of 0.3%.

### High throughput permeability assay results

**Figure 6:**
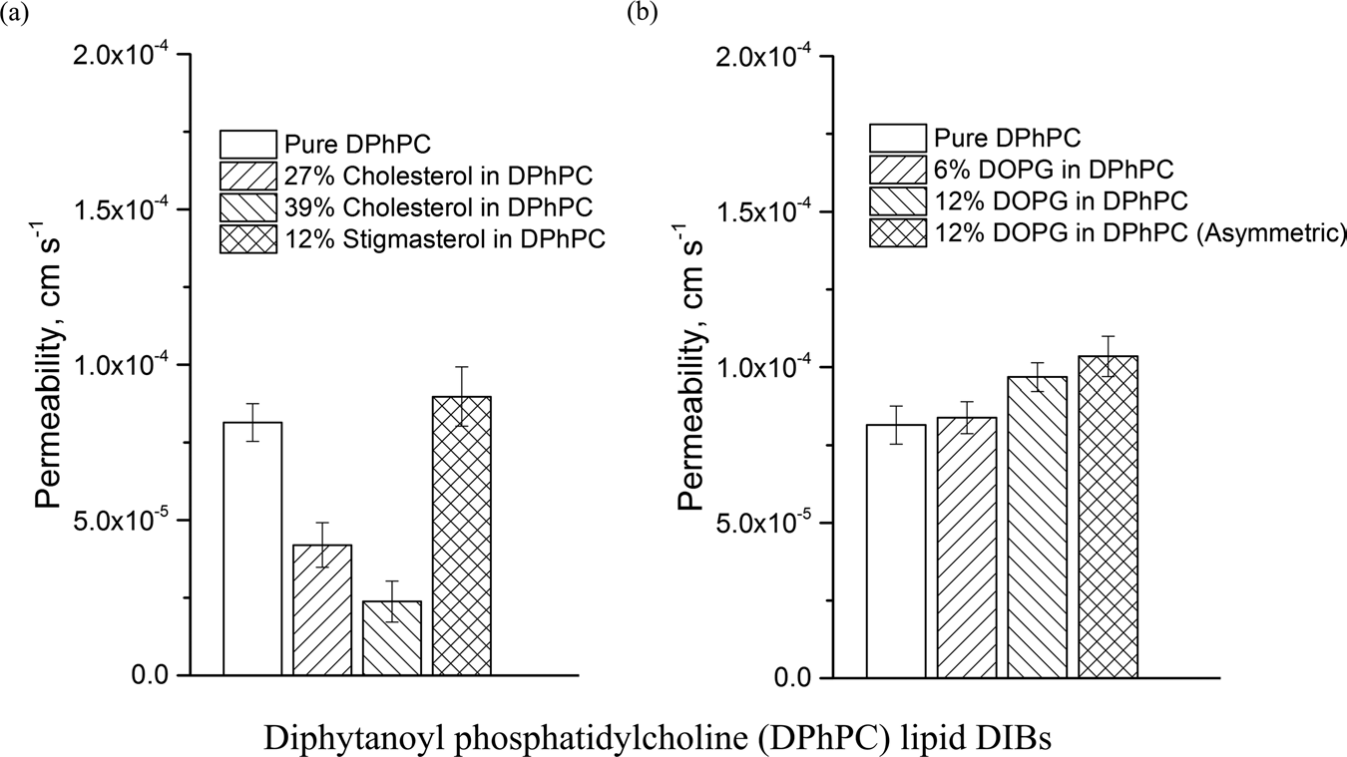
Results of the resorufin permeability assay (7.4 pH) at 50 RPM performed on the large rheo-DIB device with lipid mixtures of DPhPC and varying amounts of (**a**) cholesterol or stigmasterol and (**b**) DOPG. The sample sizes vary from 7 to 68 depending on DIB system stability and the error bars are defined as 95% confidence intervals.

**Figure 7:**
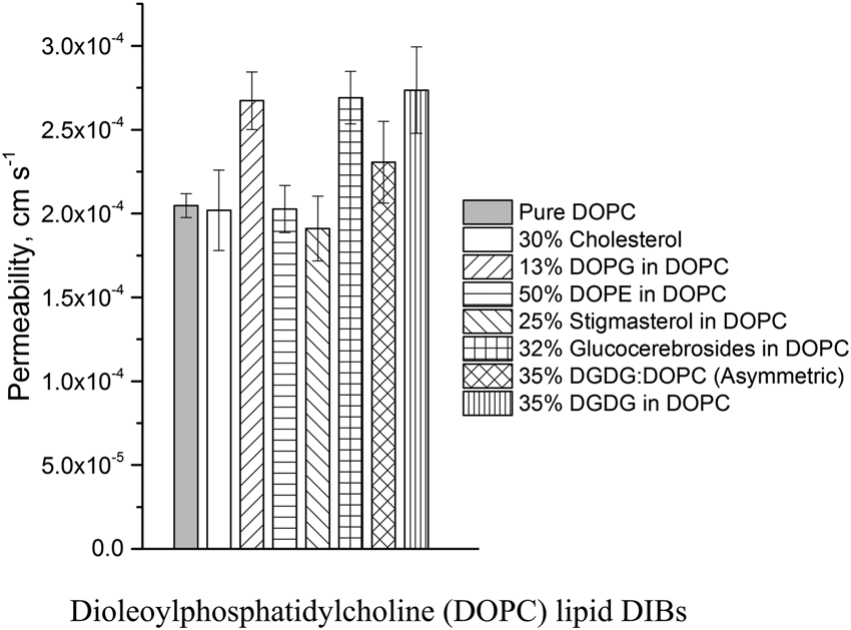
Results of the resorufin permeability assay (7.4 pH) at 50 RPM performed on the large rheo-DIB device with lipid mixtures of DOPC and varying amounts plant lipids such as DOPG, DOPE, stigmasterol, glucocerebrosides and DGDG. The sample sizes vary from 21 to 67 depending on DIB system stability and the error bars are defined as 95% confidence intervals.

The results of several permeability assays at 50 RPM are provided in Figs 6 and 7. The results consists of 1,2-diphytanoyl-sn-glycero-3-phosphocholine (DPhPC) and 1,2-dioleoyl-sn-glycero-3-phosphocholine (DOPC) based assays with varying amounts of test lipids such as 1,2-dioleoyl-sn-glycero-3-phospho-(1′-*rac*-glycerol) (DOPG), 1,2-dioleoyl-sn-glycero-3-phosphoethanolamine (DOPE), cholesterol, digalactosyldiacylglycerol (DGDG), stigmasta-5,22-dien-3-ol (stigmasterol), and glucocerebrosides. The permeability measurement is identical to that of the proof of concept device: the fluorescence intensity of the DIBs are measured for each time step over a 10 minute period, and along with the known droplet volume (0.9 µL) and interfacial surface area (0.28 mm^2^), the experimental permeation data is fit to Fick’s first law.

In this case, as the shearing rate is high, it is assumed that the UWL is negligible and that the effective permeability is a close approximation to the effective permeability. This can be verified by comparing the results of the permeability of resorufin across stirred DOPC DIBs in the large rheo-DIB device to that of the proof of concept value found by fitting experimental data to the COMSOL model. Recall that the intrinsic membrane permeability of resorufin across a DOPC DIB membrane was already found to be 1.98 ± 0.19 × 10^−4^ cm s^−1^, this compares very well with the measurement on the large rheo-DIB device at 50 RPM with a permeability measurement of 2.05 ± 0.07 × 10^−4^ cm s^−1^.

The results of the assays with the lipid DPhPC are provided in Fig. 6. The effect of cholesterol is apparent when added to DPhPC lipid DIBs, where by adding 27 and 38% cholesterol, the permeability dropped from 8.07 ± 0.62 × 10^−5^ cm s^−1^ to 4.20 ± 0.72 × 10^−5^ cm s^−1^ and 2.38 ± 0.66 × 10^−5^ cm s^−1^ respectively. Note that the stability was found to be a problem for cholesterol lipid DIBs where the survival rate was found to be 12% for high cholesterol content. This decrease in permeability is not surprising as this has already been shown for osmotic permeability for cholesterol in DPhPC lipid DIBs^43^. Indeed, there exists several examples that confirm the decrease in membrane permeability due to added cholesterol, such as in glucose permeability across dimyristoylphosphatidylcholine (DMPC) lipid vesicles or salicylic acid across DPhPC vesicles, and in molecular dynamic simulations of hypericin diffusion across dipalmitoylphosphatidylcholine (DPPC) lipid membranes^44–46^. Here the lack of an effect of membrane permeability due to the plant sterol (12% stigmasterol) is demonstrated in Fig. 6a, which shows a statistically insignificant change in permeability in the DPhPC lipid emulsion. This is coherent with the results found previously by Grunwald in a study of cellular electrolyte leakage variation due to the addition of sitosterol, stigmasterol or cholesterol^47^.

The usefulness of the rheo-DIB device is showcased in the analysis of the results of the *stagnant* permeability measurement of pure DPhPC at 5.54 ± 0.41 × 10^−5^ cm s^−1^, and 12% DOPG at 5.92 ± 0.87 × 10^−5^ cm s^−1^ respectively, where the UWL appears to wash out the difference in permeability. However for the *stirred case*, though the assays comparing pure DPhPC and with 6% DOPG lipid show little difference with permeation rates of 8.07 ± 0.62 and 8.39 ± 0.51 × 10^−5^ cm s^−1^ respectively, in Fig. 6b the addition of 12% DOPG shows a statistically significant increase in permeability at 9.68 ± 0.45 × 10^−5^ cm s^−1^, and 1.03 ± 0.64 × 10^−4^ cm s^−1^ for the asymmetric DIB case. Note that higher DOPG lipid content was not stable under shear.

Additionally, six DOPC based assays were performed with various plant lipids including cholesterol, DOPG, DOPE, stigmasterol, glucocerebrosides, and DGDG. Note that some lipid mixtures do not form stable DIBs even at low dilution, and only the highest stable lipid systems were assayed. The results in Fig. 7 not only indicate a clear distinction between the intrinsic permeability of DPhPC and DOPC, but demonstrate the permeabilizing effect of the lipids DOPG, gluococerebroside and DGDG. The addition of the lipid DOPE in DOPC was shown to have little effect on resorufin permeability (P = 2.03 ± 0.14 × 10^−4^ cm s^−1^, stability limit 50%), nor was the drop in permeability with stigmasterol (P = 1.91 ± 0.20 × 10^−4^ cm s^−1^, stability limit 25%) statistically significant. However, it was apparent that DOPG (P = 2.67 ± 0.17 × 10^−4^ cm s^−1^, stability limit 86%), glucocerebrosides (P = 2.69 ± 0.16 × 10^−4^ cm s^−1^, stability limit 32%), and DGDG (P = 2.76 ± 0.26 × 10^−4^ cm s^−1^, stability limit 35%) had the ability to permeablize the DOPC lipid membrane significantly. Interestingly, it was found that there did not appear to be a strong cholesterol dependence on DOPC permeability up to 30% cholesterol. The permeability of the cholesterol-DOPC DIBs were found to be P = 2.02 ± 0.24 × 10^−4^ cm s^−1^ with 59 samples. It is possible that at higher cholesterol content the permeability could be suppressed, however, this measurement is unfortunately limited by DIB stability.

## Discussion

As has been previously established Thomson and Dietschy, the effect of the UWL cannot be ignored with respect to measuring passive membrane permeation^15^. Indeed, as indicated by the rheo-DIB permeability assay, it is clear that the unstirred effective permeability is *ca*. 55% lower than the intrinsic membrane permeability value for the specific case of resorufin permeability in µL scale DIBs. It is worth noting that the experimental conditions adopted in this study, including permeating molecule size, droplet size and content, are similar to those reported in previous research works^3,4^. Furthermore, we have shown that this application can be applied in a high throughput manner, where we have measured the permeability of various lipids, and have shown the UWL can often obscure permeability data that is not performed in stirred conditions (when *P*_*UWL*_ is of a similar order of magnitude or lower than *P*_*m*_). Note that for very slowly membrane permeating solutes relative to the bulk diffusivity (*i*.*e*. ${P}_{UWL}\gg {P}_{m}$), the rate limiting step is the solute diffusion across the membrane. In this case mixing is not required, and the effective permeability will be equal to the intrinsic permeability. Nevertheless, we conclude that the unstirred effective permeability *P*_*eff*_, which is the parameter traditionally measured in DIB-based assay, can greatly underestimate the actual intrinsic membrane permeability *P*_*m*_. To this end, the rheo-DIBs chip is the first device that can be successfully used for measuring with a good approximation the effective and intrinsic membrane permeability together with the UWL thickness in DIBs.

More generally, as a new technology the rheo-DIB device could be broadly applied as a new tool for investigating bio-physical and bio-chemical phenomena in cell and DIBs model membranes which are known to be affected by bulk diffusion and/or shear stresses. For instance, the effect of shear stress on membrane fluidity has been previously reported as it is believed shear flows to affect lipid ordering and even modify protein activity^48–50^. In this context, little has been done on the DIB platform to investigate how shear stress affects the functionality of bio-membranes such as membrane-protein interactions, cellular transport and metabolism or mechnosensitivity^49,51^. Furthermore, this device opens the door to many new experiments relevant to important aspects of cellular biology such as mass transport and function of cardiovascular, respiratory, and gastrointestinal systems^52^. Indeed, fluid dynamic effects are not limited to mammalian cells, even plant cells are subject to fluid flow such as cytosolic streaming which is also known to control cellular function^53,54^. Furthermore, from an electrophysiology standpoint, the UWL is also shown to be of some concern as has been established that the cytoplasmic potential is “extremely” sensitive to unstirred layer thickness adjacent to cells and cell walls, as it can cause underestimates in transmembrane potential due to the increased ion flux resistance^55,56^.

## Methods

### Preparation of Lipid Emulsions

All lipids/extrusion equipment was procured from Avanti Polar lipids and the fluorescence dye 7-hydroxy-3H-phenoxazin-3-one (resorufin), bis[N,N−bis(carboxymethyl)aminomethyl]fluorescein (calcein), and cholesterol was purchased from Sigma Aldrich. Emulsions were formed from chloroform deposited lipid films and were extruded 21 times through a 0.1 nmpore in phosphate buffer (100 mM) at 5 mg mL^−1^, note that the SUVs formed were approximately 120 nm in diameter. The lipids, 1,2-dioleoyl-sn-glycero-3-phosphocholine (DOPC), 1,2-diphytanoyl-sn-glycero-3-phosphocholine (DPhPC), 1,2-dioleoyl-sn-glycero-3-phospho-(1’-*rac*-glycerol) (DOPG), 1,2-dioleoyl-sn-glycero-3-phosphoethanolamine (DOPE), digalactosyldiacylglycerol (DGDG), stigmasta-5,22-dien-3-ol (stigmasterol), and glucocerebrosides were purchased froAvanti Polar Lipids.

The fluorescent dye was added as the final step in a serial dilution at 40, 20, 10, 5, 2.5, 1.25, and 0.625 μM. The buffers used for the calcein leakage assay were 4-(2-hydroxyethyl)-1-piperazineethanesulfonic acid (HEPES) and potassium chloride (KCl). Note also that the buffer concentration is much higher than that of the resorufin, this helps to keep the droplets osmotically balanced and prevent water flux from the sink droplet to the source droplet.

### Preparation of rheo-DIB Chips

PMMA sheeting was purchased from Weatherall-UK and laser cut using a Universal Laser Systems VLS 3.60 Platform. The chip is fabricated using laser cut PMMA layers that form the stationary well plate assembly and spinning disk. The well plate assembly consists of a 1 mm thick base plate solvent (acetone) bonded to a 1 mm thick well plate, a 1 mm spacer plate, and a 6 mm overflow plate. The chambers in the well plate are cut to replicate the equilibrium shape of the DIB, which is approximated by two intersecting cylindrical chambers such that the interface diameter is ~40% of the droplet diameter. The intersecting circles are aligned along the angular axis so that the flow is directed toward the membrane. The spacer plate allows for the flow field to develop above the DIB wells. Depending on the technique, a 3 mm thick microscope plate is then bonded on the top of the overflow plate to complete well plate assembly. For the proof of concept device, a 6.3 cm diameter rotating disk is made of laser cut 1 mm thick PMMA sheet bonded to a 6 mm diameter timing belt pulley with a pair of metal radial ball bearings (2 mm ID, 6 mm OD), purchased from RS Components. The rotating disk is driving with a brushed DC motor (7.98 Watt/15 Volt). The DIBs are imaged with an Olympus IX-81 inverted microscope using a 2x objective with a TRITC fluorescence filter – excitation at 557 nm and detection at 576 nm. For the expanded device, a 14 cm diameter well plate assembly is fabricated in a similar fashion as the proof of concept device with the addition of 8 DIB wells in a radial column 7 mm apart. A 13.9 cm diameter rotating disk is made of a laser cut 1 mm thick sheet bonded to the timing belt pulley *without ball bearings*. Instead, a 3 mm diameter pin is used and lubricated with hexadecane so that the disk turns easily with minimal resistance. The device is mounted on an Olympus SZ-STL non-reflective base and imaged with a QImaging Retiga EXi Fast 1394 camera at 400 ms exposure time. The source light is filtered by a Semrock RazorEdge 532 nm short pass filter and the DIBs are illuminated directly from the side (90° from the imaging direction). The fluorescent DIB images are acquired with a 575–625 nm band pass filter. The light source consists of a fibre optic cable attached to a Stocker Yale Imagelite m20 broad spectrum light source set to maximum output. More information is provided in the ESI.

### Permeability Assay

The effective membrane permeability assay is performed as previously established, where the dynamic resorufin fluorescence intensity is fit to a single exponential decay or Fick’s first law^4,57^. The single 0.9 μLlipid emulsion droplets loaded with 0.625 and 5 µM resorufin respectively are incubated next to the rheo-DIB wells that are immersed in hexadecane for 40 + minutes to ensure complete surface coverage. Once the droplets have incubated they are set into the DIB wells and allowed to form membranes. Directly, the disk is applied to the top of the device and the DIBs are subjected to shear stress at various disk speeds as the dye permeates the membrane. The images are processed with standard techniques in the MATLAB image processing toolbox.

## Electronic supplementary material

Supplementary Information
