# Supplementary material for: Rheological Droplet Interface Bilayers (rheo-DIBs): Probing the Unstirred Water Layer Effect on Membrane Permeability via Spinning Disk Induced Shear Stress
Source: Sci Rep. 2017 Dec 14;7:17551. doi: 10.1038/s41598-017-17883-0 (PMC5730560; doi:10.1038/s41598-017-17883-0)
Supplement: Supplementary file 1 — Supplementary Information [file 41598_2017_17883_MOESM1_ESM.docx]

**Electronic Supplementary Information:**

**Rheological Droplet Interface Bilayers (rheo-DIBs): Probing the Unstirred Water Layer Effect on Membrane Permeability via Spinning Disk Induced Shear Stress**

Nathan E. Barlow,^a b^ Guido Bolognesi,^a c^ Stuart Haylock, ^a b^ Anthony J. Flemming ^b d^, Nicholas J. Brooks,^a b^ Laura M. C. Barter ^a b^ and Oscar Ces^*a b^

a. Department of Chemistry, Imperial College London, Exhibition Road South Kensington, London, UK, SW7 2AZ. E-mail: o.ces@imperial.ac.uk.

b. Institute of Chemical Biology, Imperial College London, Exhibition Road South Kensington, London, UK, SW7 2AZ.

c. Department of Chemical Engineering, Loughborough University, Loughborough, Leicestershire, LE11 3TU, UK

d. Syngenta, Jealott's Hill International Research Centre, Bracknell, Berkshire, UK, RG42 6EY

**Note on the definition of “unstirred water layer”.** As discussed in the literature^1^, the model leading to manuscript equation (6) is only an approximation of the actual system, since the concentration profile is not linear but curved (see Figure 1b and c). Nevertheless, manuscript equation (6) can still be used as an effective definition of the UWL thickness for empirical measurements, even though it does not match numerically the rigorous definition of manuscript equation (3).

**Rheo-DIB construction.** The rheo-DIB is constructed of 1 mm thick sheets of PMMA as shown in **Figure S1**. The well plate assembly (stationary) is formed by acetone bonding the 8.4 x 8.4 cm base plate, well plate, spacer and overflow plate (the final overflow plate is 6 mm thick). The well plate has various 1 mm diameter cylindrical DIB wells cut through the plate placed at 17 mm from the centre of the device. The spacer plate has a 5.4 cm hole cut through the plate which allows for the disk to rest on the edge of the spacer plate. The overflow plate has a 7.2 cm hole cut through the plate that allows room for the disk to rotate. The disk consists of a 6.3 cm diameter circular plate with a hole in the centre to accommodate a pulley, ball bearing and a 2 mm diameter pin. The disk can be removed for cleaning.

**
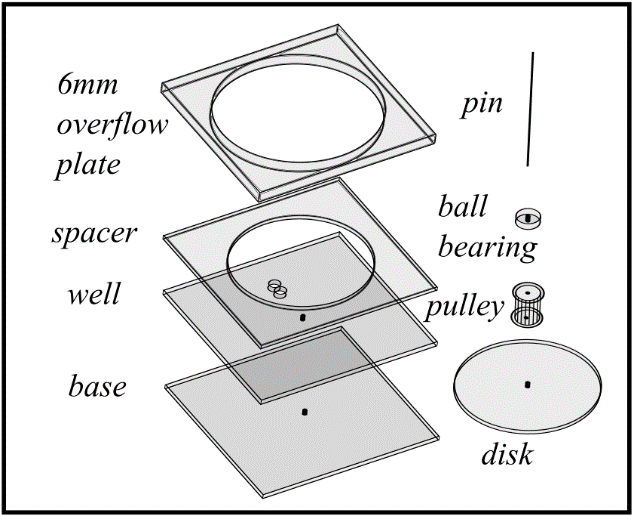
**

**Figure S1**: Cartoon of the Rheo-DIB chip assembly. The well plate and base plate are bonded with acetone to form the DIB wells and the spacer plate is added to keep the disk height constant. Finally the overflow plate is bonded to the spacer plate to prevent shearing fluid loss. The pin is bonded perpendicular to the base plate which allows for the disk to be set with ball bearing and pulley. Note the cartoon is not to scale.

**COMSOL model.** The stationary 2D COMSOL fluid dynamics model consists of the non-polar shearing phase (hexadecane), and the aqueous DIB phase **(Figure S2**). Periodic conditions are set at the inlet and outlet boundaries of the shearing phase. The flow in the shearing phase is actuated by a sliding wall, which imparts stress onto the surface of the droplets in the well and induces the aqueous fluid circulation inside the two droplets. The sliding wall velocity is given by $u_{y}\left( R,z=1mm \right)=\omega R$ with $\omega$ the disk angular velocity and $R$ the distance between the disk axis and the membrane bilayer centre (see **Figure 1e**). The no-slip boundary condition is applied to the remaining walls. The droplet circulation pattern, as demonstrated by Pozrikidis, is predicted to exhibit a biased vorticity centre towards the fluid flow above the channel.^2^ With regards to the liquid interface, the standard condition between two-phase flow is given by equation (S1a) as a function of surface stress $\tau_{ij}^{\left( 1,2 \right)}$, the gradient of the surface tension$\sigma$, and the surface curvature $\nabla\cdot\boldsymbol{n}$**,** which is effectively the Laplace pressure for the case of droplets.^3^ Additionally, the velocity$\boldsymbol{u}$ normal to the surface of the droplet is zero (S1b) whereas the tangential components of the velocities must match (S1c), (note that $I$ is the identity matrix). Note that the length of the water-oil interface is defined by the fact that the interfacial length between the adhering droplets is a factor of 0.4 of the individual droplet diameter, which sets the total length of the droplet shearing surface across the pair.

| $\left( \tau_{ij}^{\left( 1 \right)}-\tau_{ij}^{\left( 2 \right)} \right)n_{j}+\frac{\partial\sigma}{\partial x_{i}}-\sigma\left( \nabla\cdot\boldsymbol{n} \right)n_{i}=0$  $\boldsymbol{u}\cdot\boldsymbol{n}=0$  $\boldsymbol{u}^{\left( 1 \right)}\cdot(I-\boldsymbol{n n})=\boldsymbol{u}^{(2)}\cdot(I-\boldsymbol{n n})$ | (S1a)  (S1b)  (S1c) |
| --- | --- |

By neglecting the effects of the interfacial tension, the condition simplifies to a balance of stress on either side of the surface,$\tau_{ij}^{(1)}=\tau_{ij}^{(2)}$. Note that the pressure jump from the Laplace pressure effect is assumed to be constant along the oil-water interface, so the term $\frac{\partial\sigma}{\partial x_{i}}$ is null. Furthermore, due to the spherical shape of the droplet the term $\sigma\left( \nabla\cdot\boldsymbol{n} \right)n_{i}$ is constant along the oil-water interface, so it results in an additive isotropic constant pressure term in the aqueous phase domain which does not affect the solution of the Stokes equations. Boundary conditions (S1a) and (S1c) are implemented in COMSOL by setting a single-phase fluid model with a spatially dependent viscosity. Condition (S1b) is implemented by imposing a weak constraint on the oil-water interface. Similarly, the ‘impermeability’ condition is imposed on the membrane between the droplets. The droplet domains are given initial species concentrations to match experimental bulk concentrations $C^{+}$ and $C^{-}$ in **Figure S2**. The membrane between the droplet domains is given a COMSOL condition called a ‘thin diffusing boundary’, which is assigned the experimental membrane permeability value and allows diffusive flux from one droplet to the other.


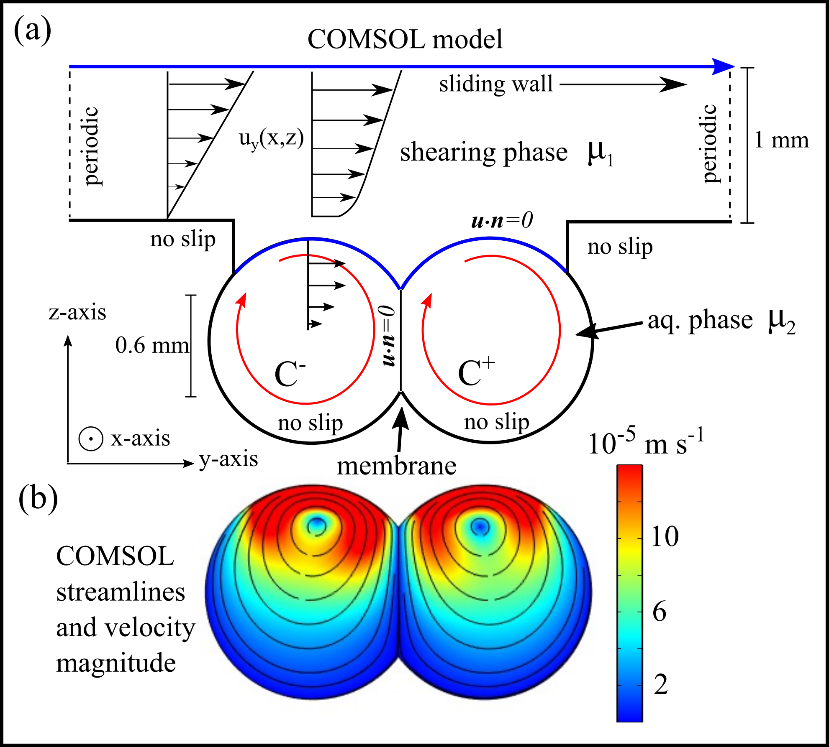


**Figure S2**: Cartoon (a) of velocity profile ($\boldsymbol{u}=u_{y},u_{z}$) in the z-y direction (no flow in the x direction) for the 2D fluid dynamic model of the rheo-DIB chip. A sliding wall (blue straight line) 1 mm above the droplets induces flow (Couette flow) in the non-polar shearing phase 1 in the y direction. This flow is well developed, periodic, and induces motion on the droplet interface (blue curved line). The moving droplet interface induces flow inside the droplet (red curved line), this causes circulation and shearing on the membrane surface. Note all black solid lines imply a no slip boundary condition, also there is no convective flux across any blue or black line ($\boldsymbol{u\cdot n}=0$), however there is species flux across the membrane based on a given membrane permeability and ‘thin layer’ thickness. Image (b) of the model result of the flow inside a DIB based on the fluid dynamics model from a shearing flow of hexadecane above the lipid encapsulated droplets. The streamlines (black lines) and the velocity magnitude show that the velocity is at the highest near the oil-water interface.

**Fitting the experimental permeability assay to the model.** The permeability assay performed on the rheo-DIB chip was fit to the 2D model by a standard least squares error method. The dimensions of the DIBs are input into the COMSOL model along with the known parameters such is water and oil viscosity, resorufin bulk diffusivity, and tangential disk speed. The initial concentration conditions are applied and a guess value for the intrinsic membrane permeability variable, $P_{m}$, is input into the model. The effective membrane permeability is measured in the same manner as the experimental technique, i.e.: the average concentration inside each droplet is calculated for each time point and is then fit to Fick’s 1^st^ law. The error in the model effective permeability and the experimental permeability is calculated and this process is repeated for various intrinsic permeability values. The $P_{m}$value with the smallest error is taken as the best fit value.

**Calcein leakage control.** It is shown that the membrane shearing does not induce poration or increased hydraulic conductivity of non-permeable molecules. A calcein-lipid solution was made with 100 mM KCl, 20 mM HEPES (pH 7.4), and 500 mM calcein. DIBs are formed of a concentrated source (+) and an empty sink (-) lipid emulsion pair. For example, the initial DIB shown in **Figure S3** exhibits an initial background fluorescence intensity, which shows no appreciable increase in intensity in the sink (-) droplet over the first 30 minutes under stagnant conditions. Furthermore, after droplets are sheared for over 2 hours at 200 RPM, no fluorescence intensity increase is apparent. If significant poration and shear induced permeability of the non-permeable membrane occurs, then fluorescence intensity in the sink (-) should increase.


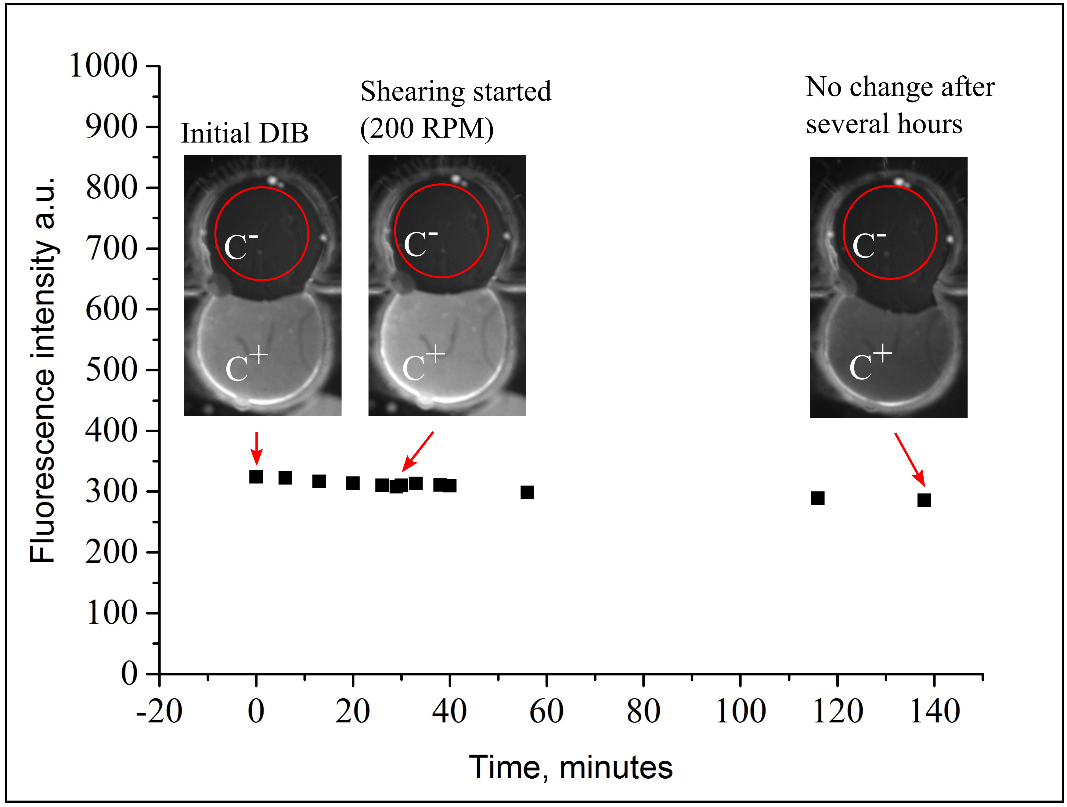


**Figure S3**: Plot of calcein leakage assay where DIBs are formed with concentrated source droplets (+) and diluted sink droplets (-) and are tested for calcein permeation with and without shearing. The source droplet (+) is significantly quenched and would increase fluorescence intensity drastically if the droplet were to leak or permeate into the sink droplet. Furthermore, the empty sink droplet also shows no appreciable increase in background fluorescence intensity. The results indicate that there is no calcein leakage into the sink droplet (-) and hence no significant membrane poration or shear induced membrane permeability of non-permeable molecules. The intensities of the sink droplets (-) are measured with imageJ from the area inside the red circles. Note that the source droplet (+) is highly quenched and the loss in fluorescence is due to photobleaching. (The image contrast is adjusted for clarity)

**Permeability assay image processing.** Micrograph images of the permeability assay is provided in Figure S4, which shows the contrast enhanced (a-f) images and original (g-l) images of the fluorescent DIBs. The dynamic fluorescence intensity is then fit to manuscript equation (7) to find the permeation rate$k$, which along with the known volume and interfacial area provides the effective permeability value$P_{\mathrm{eff}}$.


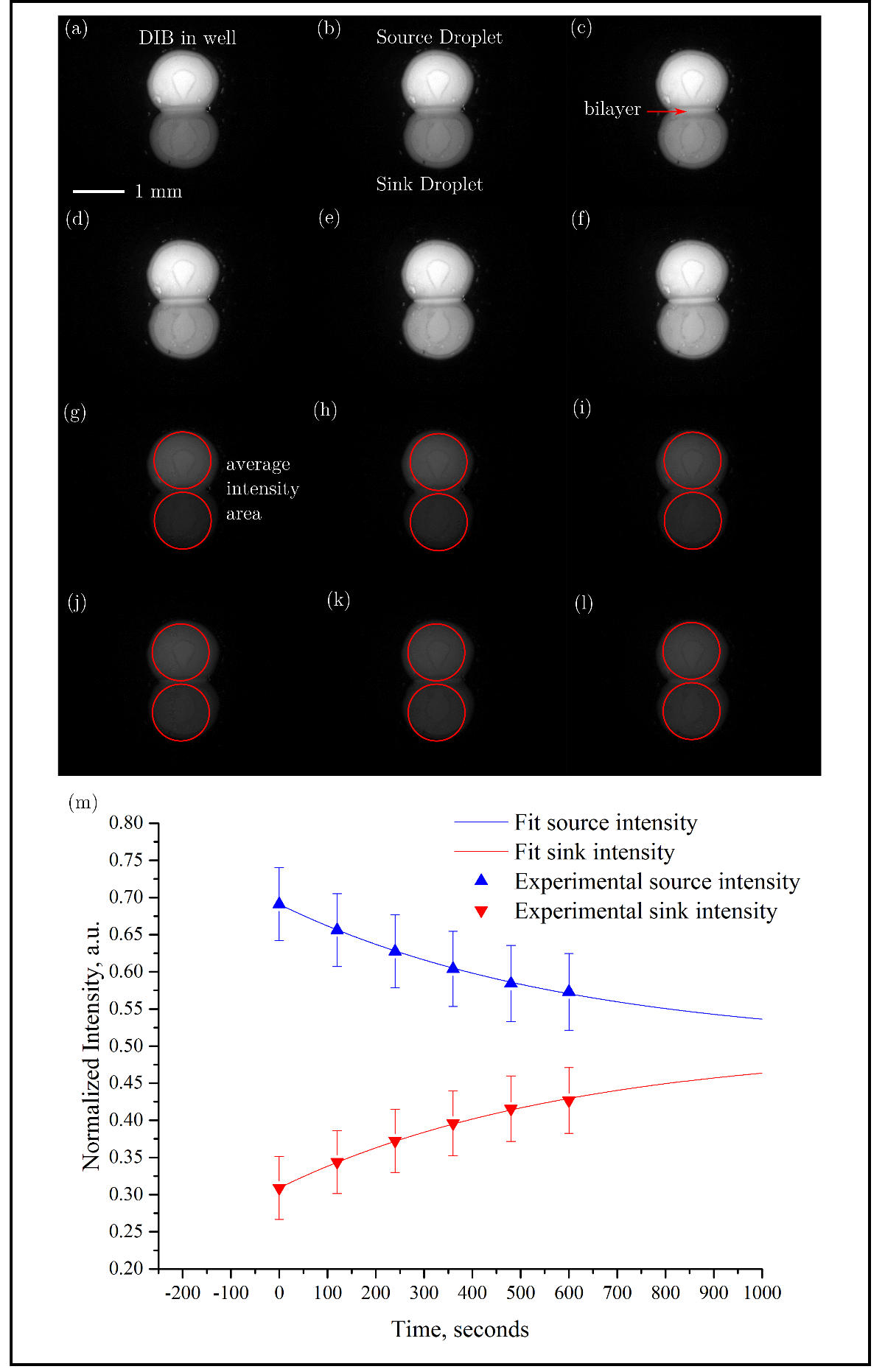


**Figure S4**: Fluorescence micrograph images of DIB permeability assay (at 30 RPM with DOPC lipids and resorufin as the permeating solute) in the rheo-DIB chip. An enhanced contrast image of the fluorescent DIBs in (a-f) show qualitatively the change in fluorescence intensity over time from [0, 2, 4, 6, 8, 10] minutes respectively. Note that the DIB conforms to the well shape and exhibits a bilayer at the interface. The original fluorescence images (g-l) are analysed for the average fluorescence intensity inside the red circles. The normalized data is then plot on (m) and fit to Fick’s first law of diffusion (note that error on the intensity is given with 1 σ standard deviation).

**Intrinsic and UWL permeability of differently charged permeants.** Resorufin is a weak acid (with a $\mathrm{pK}_{a}\sim5.8$) which exists in an aqueous solution either as an uncharged molecule H$A$ or a monoanion $A^{-}$.^4^ These species permeate the membrane at different rates and the mass balance equations for the acceptor/donor droplet pair can be hence written as follows,^5^

| $\frac{V_{d}}{A_{b}}\frac{dC_{d}}{dt}=P_{\mathrm{eff}}^{\mathrm{HA}}\left( \left[ \mathrm{HA} \right]_{a}-\left[ \mathrm{HA} \right]_{d} \right)+P_{\mathrm{eff}}^{A^{-}}(\left[ A^{-} \right]_{a}-\left[ A^{-} \right]_{d})$ | (S2a) |
| --- | --- |
| $\frac{V_{a}}{A_{b}}\frac{dC_{a}}{dt}=P_{\mathrm{eff}}^{\mathrm{HA}}\left( \left[ \mathrm{HA} \right]_{d}-\left[ \mathrm{HA} \right]_{a} \right)+P_{\mathrm{eff}}^{A^{-}}(\left[ A^{-} \right]_{d}-\left[ A^{-} \right]_{a})$ | (2Sb) |

where $V_{d}$ and $V_{a}$ are the donor and acceptor droplet volumes, $A_{b}$ is the membrane area, $P_{\mathrm{eff}}^{\mathrm{HA}}$ and $P_{\mathrm{eff}}^{A^{-}}$ are the effective permeability coefficient for H$A$ and $A^{-}$, $C_{d}=\left[ \mathrm{HA} \right]_{d}+\left[ A^{-} \right]_{d}$ and $C_{a}=\left[ \mathrm{HA} \right]_{a}+\left[ A^{-} \right]_{a}$ are the resorufin concentration in the donor and acceptor droplets. By the Henderson-Hasselbalch equation, the ratio of free acid to non-ionic species concentrations $r_{i}=\frac{\left[ A^{-} \right]_{i}}{\left[ \mathrm{HA} \right]_{i}}$ is related to the solution pH and acid dissociation constant pKa,

| $\mathrm{pH}_{i}=pK_{a}+\log r_{i}$ | (S3) |
| --- | --- |

from which

| $r_{i}={10}^{\mathrm{pH}_{i} - pK_{a}}$ | (S4) |
| --- | --- |

and

| $\frac{\left[ A^{-} \right]_{i}}{C_{i}}=\frac{r_{i}}{1+r_{i}} \mathrm{and}\frac{\left[ \mathrm{HA} \right]_{i}}{C_{i}}=\frac{1}{1+r_{i}}$ | (S5) |
| --- | --- |

with $i=d$ for the donor droplet and $i=a$ for the acceptor droplet. According to Eq. (S4) and (S5), at pH=7.5, 98% of resorufin molecules is ionic and 2% is uncharged (i.e. $r=50.1).$

If the acid dissociation reaction is fast by comparison with the rate at which the species permeate the membrane, by substituting Eq. (S5) in Eq. (S2a) and (S2b) the mass balance equations can be re-written as follows,

| $\frac{V_{d}}{A_{b}}\frac{dC_{d}}{dt}=P_{\mathrm{eff}}^{\mathrm{HA}}\left( \frac{C_{a}}{1+r_{a}}-\frac{C_{d}}{1+r_{d}} \right)+P_{\mathrm{eff}}^{A^{-}}\left( \frac{{r_{a} C}_{a}}{1+r_{a}}-\frac{{r_{d} C}_{d}}{1+r_{d}} \right)$ | (S6a) |
| --- | --- |
| $\frac{V_{a}}{A_{b}}\frac{dC_{a}}{dt}=P_{\mathrm{eff}}^{\mathrm{HA}}\left( \frac{C_{d}}{1+r_{d}}-\frac{C_{a}}{1+r_{a}} \right)+P_{\mathrm{eff}}^{A^{-}}\left( \frac{{r_{d} C}_{d}}{1+r_{d}}-\frac{{r_{a} C}_{a}}{1+r_{a}} \right)$ | (S6b) |

If donor and acceptor solutions have the same pH, then $r_{a}=r_{d}=r$

| $\frac{V_{d}}{A_{b}}\frac{dC_{d}}{dt}=\frac{1}{1+r}{( P}_{\mathrm{eff}}^{\mathrm{HA}}+r P_{\mathrm{eff}}^{A^{-}}) \left( C_{a}-C_{d} \right)$ | (S7a) |
| --- | --- |
| $\frac{V_{a}}{A_{b}}\frac{dC_{a}}{dt}=\frac{1}{1+r}{( P}_{\mathrm{eff}}^{\mathrm{HA}}+r P_{\mathrm{eff}}^{A^{-}}) \left( C_{d}-C_{a} \right)$ | (S7b) |

These equations lead to the definition of an overall effective permeability $\hat{P}_{\mathrm{eff}}$

| $\hat{P}_{\mathrm{eff}}=\frac{1}{1+r}{( P}_{\mathrm{eff}}^{\mathrm{HA}}+r P_{\mathrm{eff}}^{A^{-}})$ | (S8) |
| --- | --- |

which can be directly quantified through time-lapse fluorescence imaging of the droplet pairs. It is worth noting that the charged species $A^{-}$ is typically much less permeable than the uncharged molecules $\mathrm{HA}$ and, under the assumption $P_{\mathrm{eff}}^{A^{-}}\ll P_{\mathrm{eff}}^{\mathrm{HA}}$, Eq. (S8) can be re-written as

| $\hat{P}_{\mathrm{eff}}\simeq\frac{P_{\mathrm{eff}}^{\mathrm{HA}}}{1+r}$ | (S9) |
| --- | --- |

According to Eq.(5) in the manuscript, we can express the effective permeability $\hat{P}_{\mathrm{eff}}$ as

| $\frac{1}{\hat{P}_{\mathrm{eff}}}=\frac{1}{\hat{P}_{m}}+\frac{2}{\hat{P}_{\mathrm{UWL}}}$ | (S10) |
| --- | --- |

where $\hat{P}_{\mathrm{UWL}}$ and $\hat{P}_{m}$ are, by definition, the overall unstirred water layer permeability and intrinsic membrane permeability, respectively. Similarly,

| $\frac{1}{P_{\mathrm{eff}}^{\mathrm{HA}}}=\frac{1}{P_{m}^{\mathrm{HA}}}+\frac{2}{P_{\mathrm{UWL}}^{\mathrm{HA}}}$ | (S11) |
| --- | --- |

with $P_{\mathrm{UWL}}^{\mathrm{HA}}$ and $P_{m}^{\mathrm{HA}}$ the unstirred water layer and intrinsic membrane permeabilities for the uncharged species $\mathrm{HA}$. By replacing Eq. (S10) and (S11) in Eq. (S9), we get

| $\frac{1}{\hat{P}_{m}}+\frac{2}{\hat{P}_{\mathrm{UWL}}}\simeq(1+r)\left( \frac{1}{P_{m}^{\mathrm{HA}}}+\frac{2}{P_{\mathrm{UWL}}^{\mathrm{HA}}} \right)$ | (S12) |
| --- | --- |

For DIB-based assays at high stirring rates, the effect of the unstirred water layer is negligible (i.e. $\left( \hat{P}_{\mathrm{UWL}} \right)^{-1}\simeq\left( P_{\mathrm{UWL}}^{\mathrm{HA}} \right)^{-1}\simeq0$). It hence results from Eq. (S12)

| $\hat{P}_{m}\simeq\frac{P_{m}^{\mathrm{HA}}}{1+r}$ | (S13) |
| --- | --- |

Here we take Eq. (S13) as our definition of lumped intrinsic permeability, which takes into account the weak acid affect ($1+r$). Note that if we use this definition in Eq. (S12), we also get

| $\hat{P}_{\mathrm{UWL}}\simeq\frac{P_{\mathrm{UWL}}^{\mathrm{HA}}}{1+r}$ | (S14) |
| --- | --- |

Eq. (S13) and (S14) relate the overall permeabilities $\hat{P}_{m}$ and $\hat{P}_{\mathrm{UWL}}$, which are measured by means of the Rheo-DIB device, to the unstirred water layer and intrinsic membrane permeabilities of the most permeable species $\mathrm{HA}$.

The general trend for resorufin to become less permeable with respect to pH is demonstrated in the results shown in **Figure S5**. The stirred (50 RPM) permeability of 0.9 µL DOPC lipid DIBs are found to drop by 28% from the physiological pH of 7.4 to an alkaline condition of 8.2, and increase by 26% to an acidic condition of 6.7. More data above a pH of 8.2 and below 6.7 are impractical as DIBs become increasingly unstable.


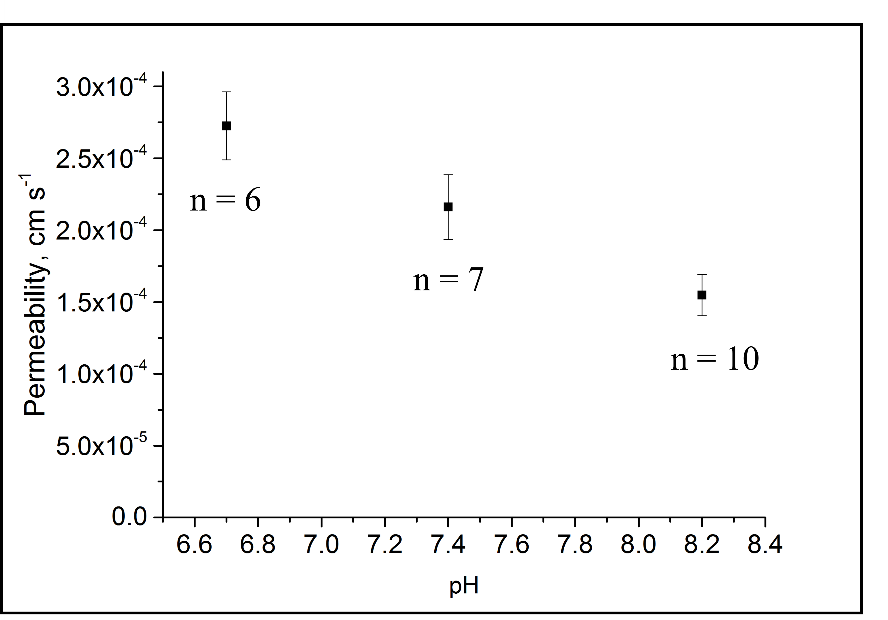


**Figure S5**: Results of stirred resorufin permeability across DOPC lipid DIBs (0.9 µL droplets) as a function of pH at 6.7, 7.4, and 8.2. The results show a decrease in permeability with respect to an increase in pH (the error bar is represented as a 95% CI).

**Membrane shear stress VS disk speed.** As shown in **Figure S6**, the shear stress $\tau\left( z \right)$ on the membrane can be measured by equation (S15) as a function of the aqueous viscosity$\mu$ and the gradient of the velocity in the z direction $\frac{\partial}{\partial y}u_{z}\left( y=0,z \right)$, where $y=0$ is the centre of the membrane.

| $\tau\left( z \right)=\mu\frac{\partial}{\partial y}u_{z}\left( y=0,z \right)$ | (S15) |
| --- | --- |

The average shear stress$\tau_{\mathrm{ave}}$ (Pa) can be found by taking the integral

| $\tau_{\mathrm{ave}}=\frac{1}{z_{\mathrm{top}}-z_{\mathrm{bottom}}}\int_{z_{\mathrm{bottom}}}^{z_{\mathrm{top}}} \tau\left( z \right)\mathrm{dz}$ | (S16) |
| --- | --- |

This is calculated from the top to the bottom of the membrane on z in COMSOL. An example of the variable shear stress along z is shown in **Figure S6** that shows the highest stress at the top of the membrane and drops close to zero at the bottom.


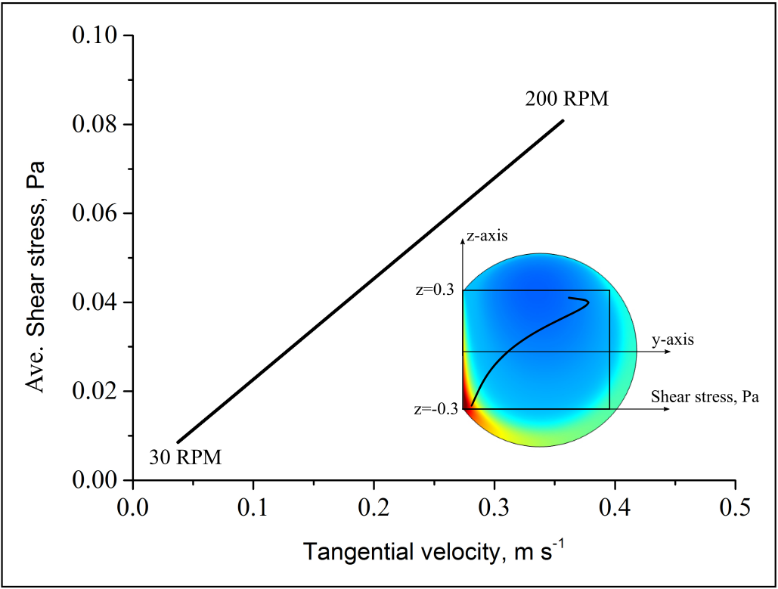


**Figure S6**: Plot of average shear stress (Pa) on the DIB membrane calculated from the 2D COMSOL model as a function of the tangential velocity of the rotating disk at R=1.7 cm from 30 to 200 RPM. The average shear stress is calculated from the variable shear stress $\tau(z)$ along the z-axis on the membrane from z = -0.3 to 0.3 mm.

1. Pedley T. Calculation of unstirred layer thickness in membrane transport experiments: a survey. *Quarterly reviews of biophysics* **16**, 115-150 (1983).

2. Pozrikidis C. Effect of membrane thickness on the slip and drift velocity in parallel shear flow. *Journal of fluids and structures* **20**, 177-187 (2005).

3. Fuller GG, Vermant J. Complex fluid-fluid interfaces: rheology and structure. *Annual review of chemical and biomolecular engineering* **3**, 519-543 (2012).

4. Coleman DJ*, et al.* A long-wavelength fluorescent substrate for continuous fluorometric determination of α-mannosidase activity: Resorufin α-d-mannopyranoside. *Analytical biochemistry* **399**, 7-12 (2010).

5. Nisisako T, Portonovo SA, Schmidt JJ. Microfluidic passive permeability assay using nanoliter droplet interface lipid bilayers. *Analyst* **138**, 6793-6800 (2013).
